# Supplementary material for: Impacts of patient advisory councils on recovery for sepsis survivors: a case study
Source: PLoS One. 2025 Oct 9;20(10):e0334057. doi: 10.1371/journal.pone.0334057 (PMC12510491; doi:10.1371/journal.pone.0334057)
Supplement: S1 Appendix — (DOCX) [file pone.0334057.s001.docx]

| No. Item | Item Description | Reported on Page # |
| --- | --- | --- |
| *Title and abstract* |  |  |
| 1a. Title and abstract | State the word “survey” along with a commonly used term in title or abstract to introduce the study’s design. | Pp. 2-4 |
| 1b. Title and abstract | Provide an informative summary in the abstract, covering background, objectives, methods, findings/results, interpretation/discussion, and conclusions. | Pp. 2-4 |
| *Introduction* |  |  |
| 2. Background | Provide a background about the rationale of study, what has been previously done, and why this survey is needed. | Pp. 4-5 |
| 3. Purpose/aim | Identify specific purposes, aims, goals, or objectives of the study. | Pp. 4-5 |
| *Methods* |  |  |
| 4. Study design | Specify the study design in the “Methods” section with a commonly used term (e.g., cross-sectional or longitudinal) | Pp. 5-6 |
| 5a. Data collection methods | Describe the questionnaire (e.g., number of sections, number of questions, number and names of instruments used). | Pp. 7-8 |
| 5b. Data collection methods | Describe all questionnaire instruments that were used in the survey to measure particular concepts. Report target population, reported validity and reliability information, scoring/classification procedure, and reference links (if any). | Pp. 7-8 |
| 5c. Data collection methods | Provide information on pretesting of the questionnaire, if performed (in the article or in an online statement). Report the method of pretesting, number of times questionnaire was pretested, number of demographics of participants used for pretesting, and the level of similarity of demographics between pre-testing participants and sample population. | Pp. 7-8 |
| 5d. Data collection methods | Questionnaire, if possible, should be fully provided (in the article, or as appendices or as an online supplement). | P 7 and S4 Appendix |
| 6a. Sample characteristics | Describe the study population (i.e., background, locations, eligibility criteria for participant inclusion on survey, exclusion criteria) | Pp. 5-7 |
| 6b. Sample characteristics | Describe the sampling techniques used (e.g., single stage or multiple stage sampling, simple random sampling, stratified sampling, cluster sampling, convenience sampling). Specify the locations of sample participants whenever clustered sampling was applied. | Pp. 6-7 |
| 6c. Sample characteristics | Provide information on sample size, along with details of sample size calculation. | Pp. 6-7 |
| 6d. Sample characteristics | Describe how representative the sample is of the study population (or target population if possible), particularly for population-based surveys. | Pp. 6-7 |
| 7a. Survey administration | Provide information on modes of questionnaire administration, including the type and number of contacts, the location where the survey was conducted (e.g., outpatient room or by use of online tools, such as Survey Monkey). | Pp. 6-7 |
| 7b. Survey administration | Provide information of survey’s time frame, such as periods of recruitment, exposure, and follow-up days. | Pp. 6-7 |
| 7c. Survey administration | Provide information on the entry process: For non-web-based surveys, provide approaches to minimize human error in data entry. For web-based surveys, provide approaches to prevent “multiple participation” of participants. | Pp. 6-7 |
| 8. Study preparation | Describe any preparation process before conducting the survey (e.g., interviewers’ training process, advertising the survey). | P 7 |
| 9a. Ethical considerations | Provide information on ethical approval for the survey if obtained, including informed consent, institutional review board [IRB] approval, Helsinki declaration, and good clinical practice [GCP] declaration (as appropriate). | P 10 |
| 9b. Ethical considerations | Provide information about survey anonymity and confidentiality and describe what mechanisms were used to protect unauthorized access. | Pp. 6-7, 10 |
| 10a. Statistical analysis | Describe statistical methods and analytical approach. Report the statistical software that was used for data analysis. | N/A |
| 10b. Statistical analysis | Report any modification of variables used in the analysis, along with reference (if available). | N/A |
| 10c. Statistical analysis | Report details about how missing data was handled. Include rate of missing items, missing data mechanism (i.e., missing completely at random [MCAR], missing at random [MAR], or missing not at random [MNAR]), and methods used to deal with missing data (e.g., multiple imputation). | N/A |
| 10d. Statistical analysis | State how non-response error was addressed. | N/A |
| 10e. Statistical analysis | For longitudinal surveys, state how loss to follow-up was addressed. | N/A |
| 10f. Statistical analysis | Indicate whether any methods such as weighting of items or propensity scores have been used to adjust for non-representativeness of sample. | N/A |
| 10g. Statistical analysis | Describe any sensitivity analysis conducted. | N/A |
| *Results* |  |  |
| 11a. Respondent characteristics | Report numbers of individuals at each stage of the study. Consider using a flow diagram, if possible. | Fig 1 |
| 11b. Respondent characteristics | Provide reasons for non-participation at each stage, if possible. | Fig 1 |
| 11c. Respondent characteristics | Report response rate, present the definition of response rate or the formula used to calculate response rate. | Pp. 6, 11 and Fig 1 |
| 11d. Respondent characteristics | Provide information to define how unique visitors are determined. Report number of unique visitors along with relevant proportions (e.g., view proportion, participation proportion, completion proportion) | P 11 and Fig 1 |
| 12. Descriptive results | Provide characteristics of study participants, as well as information on potential confounders and assessed outcomes. | Pp. 5-6, 23-24 |
| 13a. Main findings | Give unadjusted estimates and, if applicable, confounder-adjusted estimates along with 95% confidence intervals and *p* values. | N/A |
| 13b. Main findings | For multivariable analysis, provide information on the model building process, model fit statistics, and model assumptions (as appropriate), | N/A |
| 13c. Main findings | Provide details about any sensitivity analysis performed. If there are considerable amount of missing data, report sensitivity analyses comparing the results of complete cases with that of the imputed dataset (if possible). | N/A |
| *Discussion* |  |  |
| 14. Limitations | Discuss the limitations of the study, considering sources of potential biases and imprecisions, such as non-representativeness of sample, study design, important uncontrolled confounders. | Pp. 23-24 |
| 15. Interpretations | Give a cautious overall interpretation of results, based on potential biases and imprecisions and suggest areas for future research. | Pp. 21-24 |
| 16. Generalizability | Discuss the external validity of the results. | Pp. 23-24 |
| *Other sections* |  |  |
| 17. Role of the funding source | State whether any funding organization has had any roles in the survey’s design, implementation, and analysis. | Reported at submission |
| 18. Conflict of interest | Declare any potential conflict of interest. | Reported at submission |
| 19. Acknowledgements | Provide names of organizations/persons that are acknowledged along with their contribution to the research. | P 24 |
