# Supplementary material for: Impacts of patient advisory councils on recovery for sepsis survivors: a case study
Source: PLoS One. 2025 Oct 9;20(10):e0334057. doi: 10.1371/journal.pone.0334057 (PMC12510491; doi:10.1371/journal.pone.0334057)
Supplement: S3 Appendix — (DOCX) [file pone.0334057.s003.docx]

| Selection and topic | Item | Page # |
| --- | --- | --- |
| 1: Aim | *Report on aim of PPI in the study*  Despite the well-documented benefits of patient engagement and increasing prevalence of patient engagement in sepsis research and advocacy, there is limited understanding of the impacts of this involvement on sepsis survivors and their families. The primary aim of this PPI was to investigate the long-term impacts of involvement in patient advisory councils.  A secondary aim of PPI in the study was to support sepsis research that addressed the priorities of sepsis survivors and their families. The research question and study design were co-developed with members of the Patient Advisory Councils (PACs) of two sepsis research networks, and additional members were co-investigators for the study. These individuals either participated in or were consulted during data analysis and interpretation to ensure findings were aligned with patient perspectives. | Pp. 4-5 |
| 2: Methods | *Provide a clear description of the methods used for PPI in the study*  This mixed-methods study was initiated and led by two members of Sepsis Canada and Action on Sepsis PACs and a UBC Assistant Professor (all co-PIs). The study team also included the Action on Sepsis network coordinator and a PhD candidate serving on the Sepsis Canada Steering Committee. We engaged additional patient partners (n=4) to inform the development of data collection tools and conduct data analysis.  We first conducted a quantitative survey using the Patient Engagement In Research Scale. Patient partners (n=4, including the co-PIs) participated in adapting the survey tool and interpreting the results, which were used to inform the development of our interview guide. All current members (n=29) of the Sepsis Canada and Action on Sepsis PACs were invited to participate. The gender (primarily women), age (20-80 years), and geographic distribution (residents of 6 provinces) of participants were representative of the existing PACs, which includes both sepsis survivors and caregivers of individuals who experienced sepsis. A research assistant who had not previously engaged with any members of the PACs conducted 10 independent interviews and 1 focus group to gain an in-depth understanding of patient partners’ experiences. Three members of the research team reviewed the transcripts of three interviews and developed an initial coding framework. This framework was discussed with a patient partner until all coding frameworks were agreed. Coded transcripts were de-identified and shared with two patient partners and a researcher. The interpretation of data was discussed regularly until a consensus on coding was reached. | Pp. 5-10 |
| 3: Study results | *Outcomes – Report the results of PPI in the study, including both positive and negative outcomes.*  Patient partners contributed to the study in the following ways: conceptualizing and designing the study; selecting and refining data collection tools; co-developing coding framework and reviewing coded transcripts; analyzing the results through the lens of their sepsis experiences; and reviewing the manuscript to ensure that the discussion was reflective of patient priorities and perspectives. Additionally, the research assistant and patient partner co-presented a poster on this study at a conference focused on patient-oriented research in BC. | Pp. 10-21 and Tables 2-6 |
| 4. Discussion and conclusions | *Outcomes – Comment on the extent to which PPI influenced the study overall. Describe positive and negative effects.*  PPI was critical in the study as it informed all aspects of the study design. Their involvement ensured the research question remained relevant to patient partners and that potential risks to patient partners who were participants in the study were properly mitigated, as reflecting on sepsis experiences can be triggering for survivors and their family members. Patient partners had prior experience with being involved in research as study participants and as peer researchers, which allowed for a unique perspective to be included in this study, and the study team was able to support patient partners in building additional research capacity that complement their existing training and involvement with the sepsis research networks.  Engagement with PACs members through this study also allowed for the researchers to better understand the importance of patient engagement, and patient priorities and expectations in their engagement. This will allow for better partnership in future studies. | Pp. 21-24 |
| 5. Reflections/critical perspective | *Comment critically on the study, reflecting on the things that went well and those that did not, so others can learn from this experience.*  Patient partners in the study collaborated well and were excited to see their research question come to life. We were able to reflect the study findings to our own engagement with PACs members which enhanced the quality and satisfaction of this partnership. However, a challenge was communication and meeting project timelines, as patient partners had additional responsibilities and roles beyond the study. This is manageable but needs to be recognized in the planning stage. Ensuring there is a study team member who can provide dedicated logistical support and study coordination can also help with managing this challenge. | P 6 |

PPI = patient and public involvement
