# Supplementary material for: Impacts of patient advisory councils on recovery for sepsis survivors: a case study
Source: PLoS One. 2025 Oct 9;20(10):e0334057. doi: 10.1371/journal.pone.0334057 (PMC12510491; doi:10.1371/journal.pone.0334057)
Supplement: S4 Appendix — (DOCX) [file pone.0334057.s004.docx]

**Section 1: Experience with Sepsis**

1. Which of the following best described your experience with sepsis? Please select all that apply. (Options: Sepsis survivor, family member or caregiver of individual who experienced sepsis)
2. How long ago was your most recent sepsis experience? (e.g., 2 years and 3 months):

Years: ____ Months: ____

1. How many different encounters have you had with sepsis?
2. How long ago was your first sepsis experience? (e.g., 2 years and 3 months ago):

Years: ____ Months: ____

**Section 2: Experience within the Patient Advisory Councils**

1. Which research network do you belong to? (Options: Sepsis Canada, Action on Sepsis)
2. How long have you been a member? (e.g., 2 years and 3 months):

Years: ____ Months: ____

1. What type of projects have you supported in your role with the network? Please select all that apply.

(Options:

- Network Operations (e.g., Participating in a strategic planning workshop, Attending a steering committee meeting or Patient Advisory Council Meeting);
- Research Activities (e.g., Reviewing a grant proposal or manuscript, Attending lab/team meetings to learn about or inform sepsis research, Co-developing and or leading a research study with network members);
- Knowledge translation (e.g., Informing, participating in or leading sepsis public awareness campaigns; Speaking to students, health workers, researchers, or the public at educational symposia or lectures; Supporting or informing educational materials for patients on sepsis or post-sepsis care).
- Other (open-text response))

1. Since joining the Patient Advisory Council, on average how many hours per month have you spent on work related to your role on the Council?
2. Did you have prior experience serving as a patient partner for a research network or research project? (Options: Yes, No)

For the next series of questions, please think about your experience as a patient partner with Sepsis Canada or Action on Sepsis and respond to the statements by ticking only one box for each statement. If you are unsure about which option to choose for a statement, please give the best response you can.

For the purposes of these questions, a project can be any of the activities you have supported in your role with either research network. If you have taken part in multiple projects, please select the response that best captures your overall experience.

All questions have following response options:

| Strongly  Agree  **☐** | Agree  **☐** | Neutral  **☐** | Disagree  **☐** | Strongly Disagree  **☐** | Not Applicable  **☐** |
| --- | --- | --- | --- | --- | --- |

Procedural Requirements

The following fourteen (14) statements are about your general experiences throughout the project:

PR1. I was interested in the issue(s) being researched in the project

PR2. The research team members were properly introduced to each other

PR3. The number of patient partners on the research project team seemed appropriate

PR4. I understood the objective(s) of the project

PR5. I agreed with the objective(s) of the project

PR6. I understood how I could contribute to the project

PR7. I received sufficient explanation about the project

PR8. I understood my ethical responsibilities for the project

PR9. In general, I had sufficient opportunities to contribute to the project

PR10. I was able to perform my tasks for the project

PR11. I participated in making decisions about the project

PR12. I received sufficient updates about the project

PR13. Communication within the research team was clear throughout the project

PR14. The project was worth the time I spent on it

Convenience

The following four (4) statements are about how convenient it was for you to contribute throughout the project:

CN1. I had the opportunity to provide input into selecting my tasks for the project

CN2. My preferences for meetings (such as time, duration, location, and format) were considered when planning meetings

CN3. Throughout the project, I had sufficient time to complete my tasks for the project

CN4. I had opportunities to express my views

Contributions

The following four (4) statements are about your contributions throughout the project:

CT1. I contributed by providing my perspective

CT2. My contributions were a good use of my time

CT3. I shared my knowledge within the project team

CT4. My workload in the project was manageable

Team Environment and Interaction

The following five (5) statements are about the research environment and interaction throughout the project:

T1. Throughout the project, I felt accepted as a member of the research project team

T2. I was an equal partner in the research project team

T3. My interactions within the research project team were positive

T4. There was mutual respect among the research project team members

T5. There was trust among the research project team members

Support

The following three (3) statements are about the support provided throughout the project:

SU1. I received sufficient support to contribute to the project (for example, orientation, readings, training workshops, webinars)

SU2. Any concerns I had were addressed

SU3. I was offered sufficient reimbursement for my out-of-pocket expenses (such as childcare, parking, and travel) related to the project activities

Feel Valued

The following three (3) statements are about your feeling of being a valued member of the research team.

FV1. The research project team appreciated my contributions

FV2. The research project team was open to receiving my views

FV3. I was offered sufficient recognition for my contributions (for example, payment, authorship, or gifts)

Benefits

The following four (4) statements are about the benefits of your involvement in the project:

BE1. I enjoyed being a part of the project

BE2. I made an impact on the decisions in the project

BE3. I saw how my contributions could benefit others

BE4. My involvement had positive impacts on my life

Is there anything else you wish to share about your role and experiences as a member of a sepsis research network, or the impact it has had on your sepsis or post-sepsis journey? (Open-ended response)

**Section 3: Demographics**

1. Where do you currently reside? (Options: Alberta, British Columbia, Manitoba, New Brunswick, Newfoundland and Labrador, Nova Scotia, Ontario, Prince Edward Island, Quebec, Saskatchewan, Northwest Territories, Nunavut, Yukon, I prefer not to answer)
2. Which age group do you belong to? (Options: <20 years, 20-30 years, 30-40 years, 40-50 years, 50-60 years, 60-70 years, 70-80 years, 80-90 years, > 90 years, I prefer not to answer)
3. What is your gender? (Options: Woman, Man, Non-binary, Genderfluid or genderqueer, I do not know, I prefer not to answer, Another gender identity (please specify): open-text)
4. What is the highest level of education you have completed? (Options: Some high school, no diploma; High school graduate, diploma or the equivalent (for example: GED); Postsecondary (for example: Trade/technical/vocational training, Bachelors); Postgraduate (for example: Masters or Doctorate); I prefer not to answer; Other (open-text))
5. What was your employment status before your sepsis experience? (Options: Full-time employee, Part-time employee, Self-employed, Stay-at-home parent/caregiver, Retired, Unemployed, I prefer not to answer, Other (open-text))
6. Have you returned to the same employment status following your sepsis experience? (Options: Yes, No, I prefer not to answer)
7. How many months did it take you to return to the same employment status? Leave this blank if you can’t remember.
