# Supplementary material for: Impacts of patient advisory councils on recovery for sepsis survivors: a case study
Source: PLoS One. 2025 Oct 9;20(10):e0334057. doi: 10.1371/journal.pone.0334057 (PMC12510491; doi:10.1371/journal.pone.0334057)
Supplement: S5 Appendix — (DOCX) [file pone.0334057.s005.docx]

*Introduction*

Which network are you part of? What drew you to the council/network?

*Sepsis Recovery & Impact of Engagement*

1) Can you tell me about your sepsis recovery journey? What did you expect your recovery to look like?

Probes: Did you expect to be able to return to work shortly after? What impacts did you expect it to have on your physical health? Mental health? Social network? How long did you expect these impacts to take?

2) Did your participation in the council impact your recovery in any way? In what way?

Probes: How did your recovery change over the course of your involvement with the council? Were there different impacts on your physical recovery? Mental recovery? Social support system? Were there factors in the way the council operates that impeded or supported your recovery?

3) Can you tell me about your experience participating in the patient council?

Probes: What types of activities did you participate in; how long have you been involved in the council, how much time do you commit on the average week/month/year? How frequent are the related activities and meetings? How was the council organized? What type of support were you provided with? What type of recognition/compensation do you receive?

*Experience with the Council*

4) Can you tell me about your expectations for involvement in the council? What did you expect your participation would look like before you started? Did it meet these expectations - how or how not?

Probe: Did you have a role in decision-making? Did you expect more or less support? Did you expect more or less opportunities to share your perspective? What knowledge or information did you contribute?

5) What motivated you to join the council? Do you feel your contributions are valued?

Probes: What made you feel this way? Did this change over time or depend on the specific activity you were involved in? Did this change depending on the people who worked with?

6) What made participating in the council easy and/or challenging?

Probes: Did the meeting times interfere with other commitments or time constraints? Did you have input into when meetings occurred? Did other team members provide support? Were you provided with resources or training to support your participation? Did the format of the meeting include opportunities to contribute?

*7*) Did you form any new personal or professional relationships as a result of your involvement in the council?

If yes - Did these relationships extend to activities outside of those organized or supported by the council? What impact did these relationships have on your recovery from sepsis?

8) Do you think your participation in the council impacted others' recovery from sepsis?

Probes: For example, what about your family members? Other council members? General public?

9) What impact do you think your involvement with the council will have on your recovery in the future?

Probes: Do you think it will impede your recovery, support it, or have no effect? Why or why not?

10) Would you recommend other sepsis survivors/family members of sepsis survivors participate in patient advisory councils? Why/why not?

Probes: Are the activities or time and energy commitment appropriate? Would they agree with the objectives? Do you think they could benefit from the experience? Has the experience with other members been positive or negative?

*Closing*

11) If you could make changes to the way the council operates, what would you recommend?

Probes: activities, objectives, time/energy commitment, processes for recruiting or engaging with members, support provided, recognition provided

12) How would you recommend we share lessons learned from this study with yourself and other patient partners in the future? Probe: Format (i.e., presentation vs online resource vs brief report)
